# gD-Independent Superinfection Exclusion of Alphaherpesviruses

**DOI:** 10.1128/JVI.00089-16

**Published:** 2016-03-28

**Authors:** A. Criddle, T. Thornburg, I. Kochetkova, M. DePartee, M. P. Taylor

**Affiliations:** Department of Microbiology and Immunology, Montana State University, Bozeman, Montana, USA; University of California, Irvine

## Abstract

Many viruses have the capacity to prevent a cell from being infected by a second virus, often termed superinfection exclusion. Alphaherpesviruses, including the human pathogen herpes simplex virus 1 (HSV-1) and the animal herpesvirus pseudorabies virus (PRV), encode a membrane-bound glycoprotein, gD, that can interfere with subsequent virion entry. We sought to characterize the timing and mechanism of superinfection exclusion during HSV-1 and PRV infection. To this end, we utilized recombinant viruses expressing fluorescent protein (FP) markers of infection that allowed the visualization of viral infections by microscopy and flow cytometry as well as the differentiation of viral progeny. Our results demonstrated the majority of HSV-1- and PRV-infected cells establish superinfection exclusion by 2 h postinfection. The modification of viral infections by virion inactivation and phosphonoacetic acid, cycloheximide, and actinomycin D treatments indicated new protein synthesis is needed to establish superinfection exclusion. Primary infection with gene deletion PRV recombinants identified that new gD expression is not required to establish superinfection exclusion of a secondary viral inoculum. We also identified the timing of coinfection events during axon-to-cell spread, with most occurring within a 2-h window, suggesting a role for cellular superinfection exclusion during neuroinvasive spread of infection. In summary, we have characterized a gD-independent mechanism of superinfection exclusion established by two members of the alphaherpesvirus family and identified a potential role of exclusion during the pathogenic spread of infection.

**IMPORTANCE** Superinfection exclusion is a widely observed phenomenon initiated by a primary viral infection to prevent further viruses from infecting the same cell. The capacity for alphaherpesviruses to infect the same cell impacts rates of interviral recombination and disease. Interviral recombination allows genome diversification, facilitating the development of resistance to antiviral therapeutics and evasion of vaccine-mediated immune responses. Our results demonstrate superinfection exclusion occurs early, through a gD-independent process, and is important in the directed spread of infection. Identifying when and where in an infected host viral genomes are more likely to coinfect the same cell and generate viral recombinants will enhance the development of effective antiviral therapies and interventions.

## INTRODUCTION

Superinfection exclusion occurs when the first virus to infect a cell prevents subsequent viruses from further infecting that same cell. The extent of exclusion ranges from preventing other viruses of the same strain (autologous exclusion) to more distantly related or unrelated viruses (heterologous exclusion) from coinfecting the cell. Superinfection exclusion may protect limited cellular resources and promote the replication and dissemination of the originally infecting virus. Superinfection exclusion first was observed in bacteriophages (1) and now has been observed for a wide range of viruses, including influenza virus (2), poxviruses (3, 4), flaviviruses (5, 6), alphaviruses (7), and most importantly for this work, alphaherpesviruses.

Alphaherpesviruses are a family of neuroinvasive herpesviruses, including the human pathogen herpes simplex virus 1 (HSV-1) and the porcine herpesvirus pseudorabies virus (PRV). These viruses infect peripheral mucous membranes and invade sensory neurons, establishing lifelong, latent infections in their respective hosts (8). Despite many similarities in virion structure, infectious cycle, and pathogenesis, HSV and PRV are divergent viruses with various homologies across functionally conserved viral genes (9, 10). This dissimilarity is useful in identifying conserved functions between divergent herpesviruses through comparative analysis of the two viruses' properties (11).

The capacity for alphaherpesviruses to infect the same cell impacts rates of interviral recombination and disease. Alphaherpesviruses are ubiquitous in vertebrate species, with upwards of 80% of the human population exposed to HSV-1 (12). Given the prevalence of infection, any one individual likely is exposed to multiple HSV-1 strains throughout their lifetime. Coinfections between HSV-1 strains is a major driver of recombination-mediated diversification *in vivo*, with similar effects seen between other alphaherpesviruses (13–15). The relevance of viral superinfection exclusion to *in vivo* infections has yet to be established (16, 17). Infections of trigeminal neurons can be dominated by a single viral species, which resist later challenges by a subsequent virus (16). On the contrary, the viruses shed by individuals can vary over time, with certain patients presenting with recurrent herpetic lesions elicited from a mixed viral population, presumably through sequential exposures (18). It is currently unknown where coinfection occurs in infected hosts, at the neuronal sites of latency or at peripheral mucosal sites following reactivation. In recently published work, we identified a restriction on HSV and PRV coinfection following axon-to-cell spread (19). At that time, we hypothesized a role for superinfection exclusion in reducing the opportunity for viral coinfection.

A common molecular mechanism that mediates superinfection exclusion is receptor interference. Receptor interference occurs when viral entry proteins interact with receptors on the cell surface, thereby blocking incoming viruses from entering the cell. The majority of alphaherpesviruses encode a membrane-bound glycoprotein, gD, expressed with early-late kinetics during viral infection, although the absolute timing of gD expression is complicated (20, 21). Virion-associated gD interacts with cellular proteins, including nectin-1, to trigger virion membrane fusion, while newly synthesized gD uses the same interactions to directly interfere with subsequent virion entry or by inducing the endocytosis of cell surface receptors (22–24). Early publications concluded that cells expressing HSV-1 gD resist superinfection by a divergent group of alphaherpesviruses, including HSV, PRV, and equine herpesvirus (EHV) infection (25). Similar studies expressing gD from animal herpesviruses, including EHV and bovine herpesvirus, report similar results (26–28). Further studies identified heterologous superinfection exclusion between HSV and varicella zoster virus (VZV) during infection of neurons (29). In many of these studies it was presumed that gD-mediated receptor interference follows gD expression kinetics, establishing superinfection exclusion relatively late, between 4 and 6 h postinfection. In fact, the timing of superinfection exclusion varies greatly. Utilizing PRV recombinants expressing fluorescent protein (FP) reporters, Banfield et al. reported a reduction of FP expression associated with the lagging virus beginning 2 h postinfection in dorsal root ganglion neuron cultures (30). For bovine herpesvirus, a reduction in recombination mutants between coinfecting viruses was observed between 2 and 8 h postinfection following application of the first inoculating virus (26).

Taking disparate observations in the timing of viral exclusion into account, we sought to understand the timing and nature of herpesvirus superinfection exclusion during HSV-1 and PRV infection. We then wanted to identify the possible functioning of this exclusion mechanism during transneuronal spread of infection. Our strategy uses FP-expressing recombinants of HSV-1 and PRV in conjunction with differential inoculation times, chemical inhibitors of viral replication, and mutant viruses to characterize viral superinfection. Parallel experimentation using a cultured neuronal infection model visualized the transmission of fluorescently labeled virus particles in order to characterize the timing of viral coinfection events during axon-to-cell spread.

## MATERIALS AND METHODS

### Cells and viruses.

African green monkey kidney cells (Vero) and porcine kidney epithelial cells (PK15) were maintained in Dulbecco's modified Eagle's medium (DMEM) supplemented with 10% (vol/vol) fetal bovine serum (FBS) and 1% (vol/vol) penicillin-streptomycin. PK15 cells stably expressing gD (G5) cells (31) were maintained in the same solution supplemented with histidinol (50 μg/ml) to maintain selection. Primary cultures of rat superior cervical ganglion (SCG) neurons were maintained in neuronal medium, which consists of neurobasal medium supplemented with 1% penicillin-streptomycin-glutamine, B27 supplement, and 50 ng/ml neuronal growth factor 2.5S (NGF) (Life Technologies, Carlsbad, CA), as described in reference 19.

Vero cells were used to propagate and determine titers of all HSV-1 strain 17-derived recombinants. PK15s were used to propagate and determine the titer of all PRV Becker-derived viral recombinants. HSV-1 strains OK11 (mCherry with a triplicate nuclear localization sequence (3xNLS) and OK12 (eYFP-3xNLS) and PRV strains 286 (mCherry-3xNLS) and 287 (enhanced yellow fluorescent protein [eYFP]-3xNLS) have been described previously (19). HSV-1 MT01 and PRV 289, both expressing Turq2-3xNLS, were derived as follows. The open reading frame (ORF) for Turquiose2 fluorescent protein (32) was cloned into the OK11 fluorophore expression cassette, replacing mCherry. The resulting Turq2-3xNLS sequence expression construct, termed pMT06, was linearized by restriction digest and cotransfected with either HSV OK11 or PRV 286. Cells expressing blue fluorescent proteins were isolated and subjected to three rounds of plaque purification. Turq2-expressing recombinant viruses were compared to their cognate YFP and mCherry-expressing partners in single-step growth curves.

PRV mutants lacking gD expression were derived from the PRV bacterial artificial chromosome (PRV BAC). PRV GS442 was the kind gift of the Enquist laboratory. Briefly, GS442 is a gD insertion mutant resulting in gD promoter-driven expression of a diffusible green fluorescent protein (GFP) instead of gD (33). PRV GS6127 was a kind gift of G. A. Smith. Briefly, the gD gene was excised through homologous recombination to the PRV BAC (34). For both GS442 and GS6127, BAC DNA was purified and transfected into G5-complementing cells (31). Virus was propagated on a mixture of G5 and PK15 cells (approximately 3:1 ratio). Viral titers were determined on PK15 cells, resulting in small but visible plaques. G5 cells could not support the titering and propagation of complemented gD-null virus, presumably through previously described gD inhibition of viral entry (25).

PRV 427 is a previously described (19) recombinant expressing two fluorescent proteins. The minor capsid protein is fused to monomeric red fluorescent protein (mRFP) and is expressed from the endogenous locus, while a YFP fused to a CAAX motif is expressed from the cytomegalovirus (CMV) promoter from the gG insertion site (35).

### Microscopy and flow cytometry.

Epifluorescence imaging was performed on a Nikon Ti-Eclipse (Nikon Instruments, Melville, NY) inverted microscope equipped with a SpectraX LED (Lumencor, Beaverton, OR) excitation module and fast-switching emission filter wheels (Prior Scientific, Rockland, MA). Fluorescence imaging used paired excitation/emission filters and dichroic mirrors for cyan fluorescent protein (CFP), YFP, and RFP (Chroma Technology Corp., Bellow Falls, VT). All images were acquired using a Plan Fluor 20× phase contrast (Ph) objective and an iXon 896 EM-CCD (Andor Technology Ltd., Belfast, Northern Ireland) camera using NIS Elements software. Infected cells were imaged between 6 and 8 h postinfection, depending on virus and cell type. Three technical replicate infections were imaged per condition tested. All imaging experiments were performed a minimum of two times.

Flow cytometry of FP expression in infected cells was performed using a BD LSR II or BD LSR Fortessa (BD Biosciences, San Jose, CA). Infected cells were harvested by trypsinization, washed 1× in complete DMEM and then in Ca2^+^- and Mg2^+^-free phosphate-buffered saline (PBS), and resuspended in fluorescence-activated cell sorting (FACS) buffer (Ca2^+^- and Mg2^+^-free PBS with 0.1% bovine serum albumin [BSA]). Acquisition and gating were set on mock-infected and single-color infected cell populations. All cytometry data were analyzed using the FlowJo data analysis software (FlowJo, LLC, Ashland, OR).

### Chemical modification of primary infection. (i) UV irradiation.

Primary viral inoculum was inactivated by exposure to UV irradiation (36). One-milliliter aliquots of PRV 289 or HSV MT06 were put into a 6-cm plastic dish and subjected to approximately 2.5 × 10^5^ mW/cm^2^ of UV irradiation in a UV transilluminator (Stratalinker). The titers of irradiated viral stocks were determined for PFU content and found to be reduced by >10^5^ (approximately 1 × 10^3^ PFU/ml remaining). Volumes of irradiated inocula equivalent to a multiplicity of infection (MOI) of 10 (based on preirradiation titering) were used as the primary inocula in experiments.

### (ii) Neutral red inactivation.

Similar to UV irradiation, neutral red has been documented to inactivate the infectious capacity of herpesviruses (37). Briefly, competent viral stocks are mixed one to one with a 60 μg/ml stock of neutral red solution (from 60 mg/ml stock diluted in PBS; final concentration of 30 μg/ml). Virus/neutral red solution was incubated on ice for 30 min. Following incubation, virus/neutral red solution is placed on a color-corrected white light box (Adorama, New York, NY) for 15 min at room temperature. Inactivated viral stocks were frozen prior to use in experiments or for the analysis of remaining viral infectivity through titering.

### (iii) PAA, CHX, and ActD.

Following primary inoculation, infected cells were washed and fed with viral medium (2% FBS, 1% penicillin-streptomycin, DMEM) that was either mock treated or treated with 400 μg/ml of phosphonoacetic acid (PAA), 100 μg/ml cycloheximide (CHX), and/or 1 μg/ml actinomycin D (ActD) (Sigma-Aldrich, St. Louis, MO). All compounds were prepared as 1,000× stock solutions in water and dimethyl sulfoxide (DMSO), respectively. Infection was allowed to proceed for 2 h, followed by medium removal, PBS washing, and replacement with fresh media. The effectiveness of PAA treatment was demonstrated by harvesting treated cells (without washout) at 10 h postinfection. Titering revealed a >10^3^ PFU/ml loss of viral progeny in treated cells compared to the level for untreated controls.

### Western blot analysis.

Infected cells were harvested by scraping and pelleted at low speed, and cytosolic extracts were made with an NP-40 extraction buffer (10 mM HEPES, pH 8, 0.5% NP-40, 1.5 mM MgCl_2_, 10 mM KCl, 0.5 mM dithiothreitol [DTT], 200 mM sucrose) (38). Protein concentration was measured by bicinchoninic acid (BCA) protein quantification, and 40 μg of total protein per lane was separated on 10% SDS-PAGE and transferred to a polyvinylidene difluoride (PVDF) membrane. Immunoblotting for PRV gD with rabbit antiserum at 1:3,000 and PRV VP5 with mouse purified IgG at 1:5,000 (a kind gift of the Enquist laboratory) was performed, followed by the addition of anti-rabbit-horseradish peroxidase (HRP) and anti-mouse HRP, respectively (Santa Cruz Biotechnology, Inc., Dallas, TX), with subsequent visualization of antibody binding by chemiluminescence.

### qRT-PCR of immediate-early gene transcripts.

Quantitative real-time PCR (qRT-PCR) procedures and primers were adapted from previously published work (39). Briefly, cells were infected and treated with chemical inhibitors as described previously. At 2 and 4 h postinfection, cells were harvested by scraping and RNA was extracted using the Ambion PureLink RNA minikit (Life Technologies). Purified RNA was converted to cDNA using a Moloney-murine leukemia virus reverse transcriptase polymerase. One hundred nanograms of cDNA then was used in a qRT-PCR using PowerUp SYBR green master mix (Life Technologies). PCR primer pairs for EP0 (PRV) (39) and ICP0 (HSV) (40) as well as 28S rRNA were used to compare relative amounts of viral transcript across all conditions.

### Time-lapse imaging of axon-to-cell spread.

Compartmentalized neuronal cultures were constructed as previously described (19, 41). A three-compartment Teflon ring was mounted with sterile grease onto an optical plastic dish (Ibidi, Martinsried, Germany). Dissociated superior cervical ganglia (SCGs), dissected from day 17 rat embryos, were plated in the far compartment. Axons extend from the SCG cell bodies beneath two physical barriers, penetrating into the far compartment (see Fig. 5A). One day prior to infection, approximately 1 × 10^4^ PK15 cells were sparsely seeded onto isolated axons in the far compartment. PRV 427 inoculum (10^6^ PFU/100 μl) was applied to the SCG cell body compartment, resulting in the confluent infection of neurons. The imaging of fluorescently labeled virion transmission and expression of fluorescent markers of infection was performed in regions of the axon compartment. Isolated axons and cells were subjected to sequential-phase YFP and RFP fluorescence observation using a 60× Plan Fluor Ph objective (Nikon) every 5 or 15 min for 18 h beginning at 5 h after neuronal cell body infection. Cultures were maintained at 37°C in a 5% CO_2_ enriched atmosphere using a stage-top incubator system (Live Cell Instrument, Seoul, South Korea).

## RESULTS

To characterize the timing and potential mechanism of superinfection exclusion, we utilized HSV-1 and PRV recombinant viruses expressing FP markers of infection. FP expression allows us to visualize viral infections in single cells by microscopy or flow cytometry or to analyze progeny virus by their fluorescent protein expression.

**FIG 1:**
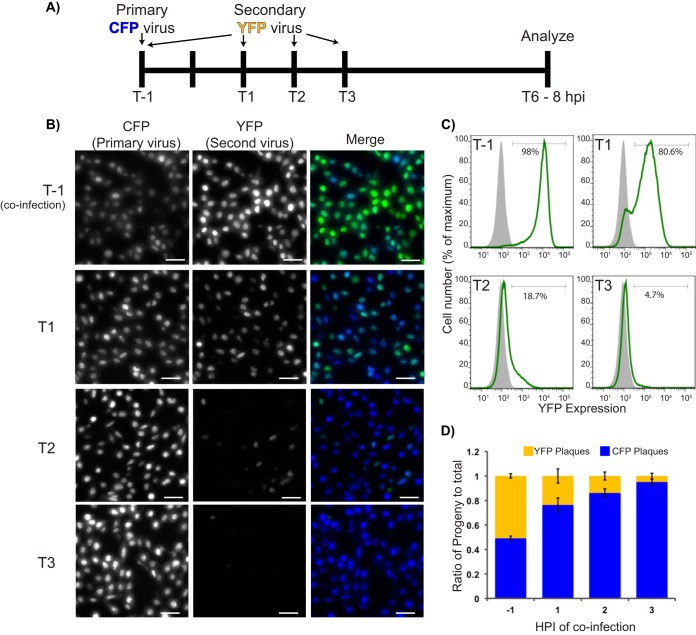
HSV superinfection exclusion. (A) A diagram of the timeline of experimentation. CFP-expressing virus (termed primary virus) is added at *T*−1. YFP-expressing virus (termed the second virus), is either added at the same time, or applied at later times postinfection (*T*1, *T*2, or *T*3). Cells then are analyzed for FP expression between 6 and 8 h postinfection (hpi). (B) Fluorescent micrographs of infected Vero cells. Cells were infected at the time points indicated in panel A at an MOI of 10 of either HSV OK12 or HSV MT01. At 8 hpi, FP expression was imaged with identical exposure settings under ×200 magnification (the scale bar is 50 μm). CFP and YFP channels are monochrome, while the two-channel merged image is in color (CFP in blue, YFP in green). Experiments were performed twice with triplicate samples for each condition. Representative images are presented. (C) Flow cytometry of YFP expression in populations of HSV-1-coinfected cells. Coinfected populations of Vero cells were tracked for YFP expression (green-line histogram) compared to in mock-infected cells (filled gray histogram). A total of 100,000 events were collected per sample. Representative data from duplicate experiments are depicted. (D) Coinfected cells were harvested at 10 hpi, and progeny virus was subjected to limiting dilution. Plaques were visualized and scored for FP expression. Replicates of three were performed per condition, with a minimum of 100 plaques counted per sample. The ratio of YFP and CFP to total plaques counted is displayed.

Using FP expression as a marker of infection, we tested when an infected cell becomes refractory to superinfection. To identify the timing of superinfection exclusion for HSV-1, we first used the HSV recombinant MT01, expressing Turq2-NLS, or OK12, expressing eYFP-NLS, to infect Vero cells. Cells were either coinoculated with MT01 and OK12 (1 h prior to infection [*T*−1]) or were inoculated with MT01 and subsequently inoculated with OK12 at 1-h intervals after the initial infection (*T*1, *T*2, and *T*3) (Fig. 1A). For consistency, the application of virus is considered 1 h prior to infection (*T*−1), whereas the removal of inoculum and application of fresh medium is considered time zero (*T*0). Inoculations of both viruses were performed at a multiplicity of infection (MOI) of 10 to ensure the majority of cells were exposed to infectious particles. Infected cells then were analyzed for fluorescent protein expression by microscopy or flow cytometry at 6 to 8 h after initial infection (Fig. 1B and C, respectively). Alternatively, infected cells were harvested and lysed and progeny virions were separated, limiting titration for quantification based on FP expression in isolated plaques (Fig. 1D). In fluorescent micrographs we see most cells express both FP markers of OK12 and MT01 infection under *T*−1 and *T*1 conditions of coinfection. At *T*2 we saw a dramatic decrease in the number of YFP-positive nuclei in micrographs and an increase in the relative intensity of CFP expression. Flow cytometry analysis of *T*−1 coinfections found 98% of the population was YFP positive. By delaying the inoculation of HSV OK12 for 2 h after initial infection, >80% of the population did not express detectable levels of YFP. Control infections of OK12 alone at the same times of inoculation demonstrated robust YFP expression and detection (data not shown). The absence of YFP expression following a 2-h delayed inoculation correlated with decreasing YFP-expressing viral progeny (Fig. 1D). Approximately 10% of the population of plaques expressed YFP at *T*2 infection. By 3 h postinfection almost 98% of viral progeny expressed CFP. Our experiments demonstrate that a secondary inoculum, delayed 2 h after initial infection, is excluded from the majority of cells based on the expression of the FP or production of viral progeny. From these results, we conclude the majority of HSV-1-infected cells establish superinfection exclusion by 2 h postinfection.

**FIG 2:**
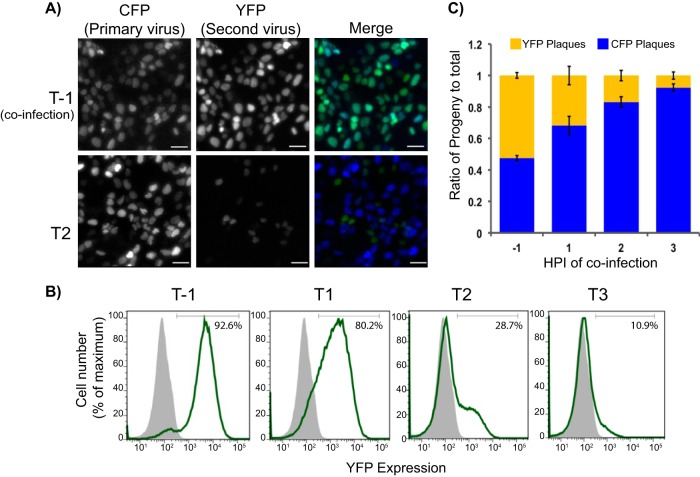
PRV superinfection exclusion. (A) PRV 287 and 289 were used to coinfect PK15 cells at an MOI of 10 for each virus under the conditions indicated. FP expression was imaged with identical exposure settings under ×200 magnification (the scale bar is 50 μm). CFP and YFP channels are monochrome, while the two-channel merged image is in color (CFP in blue, YFP in green). Experiments were performed twice with triplicate samples for each condition. (B) Flow cytometry of YFP expression in populations of PRV-coinfected cells. Coinfected populations of PK15 cells were tracked for YFP expression (green-line histogram) compared to mock-infected cells (filled gray histogram). A total of 100,000 events were collected per sample. (C) Coinfected cells were harvested at 10 hpi, and progeny virus was subjected to limiting dilution. Plaques were visualized and scored for FP expression. Replicates of three were performed per condition, with a minimum of 100 plaques counted per sample. The ratio of YFP and CFP to total plaques counted is displayed.

To determine if superinfection exclusion is a general principle of alphaherpesvirus infection, we performed similar experimentation with FP-expressing PRV recombinants. The recombinants PRV 289 (expressing a Turq2-NLS FP) and PRV 287 (expressing an eYFP-NLS FP) were used for infections of porcine kidney cells (PK15s) using conditions similar to those described for HSV-1 coinfections. Using these infection conditions, the majority of cells fail to express YFP associated with secondary, PRV 287 viral inoculum following a delay of 2 h after the primary infection (Fig. 2A and B). Both micrographs and flow cytometry displayed a reduction in YFP-positive cells following inoculation at 2 h postinfection. Progeny virus titration also demonstrated reductions in YFP-positive plaques at 2 h postinfection, similar to our observations with HSV-1. The extent and timing of YFP expression and production of viral progeny during PRV infection is similar to that seen for HSV coinfections, although the extent of exclusion at 2 h postinfection is slightly reduced. Overall, the PRV experiments demonstrated that superinfection exclusion is established by distantly related alphaherpesviruses independent of cell type and with similar kinetics.

**FIG 3:**
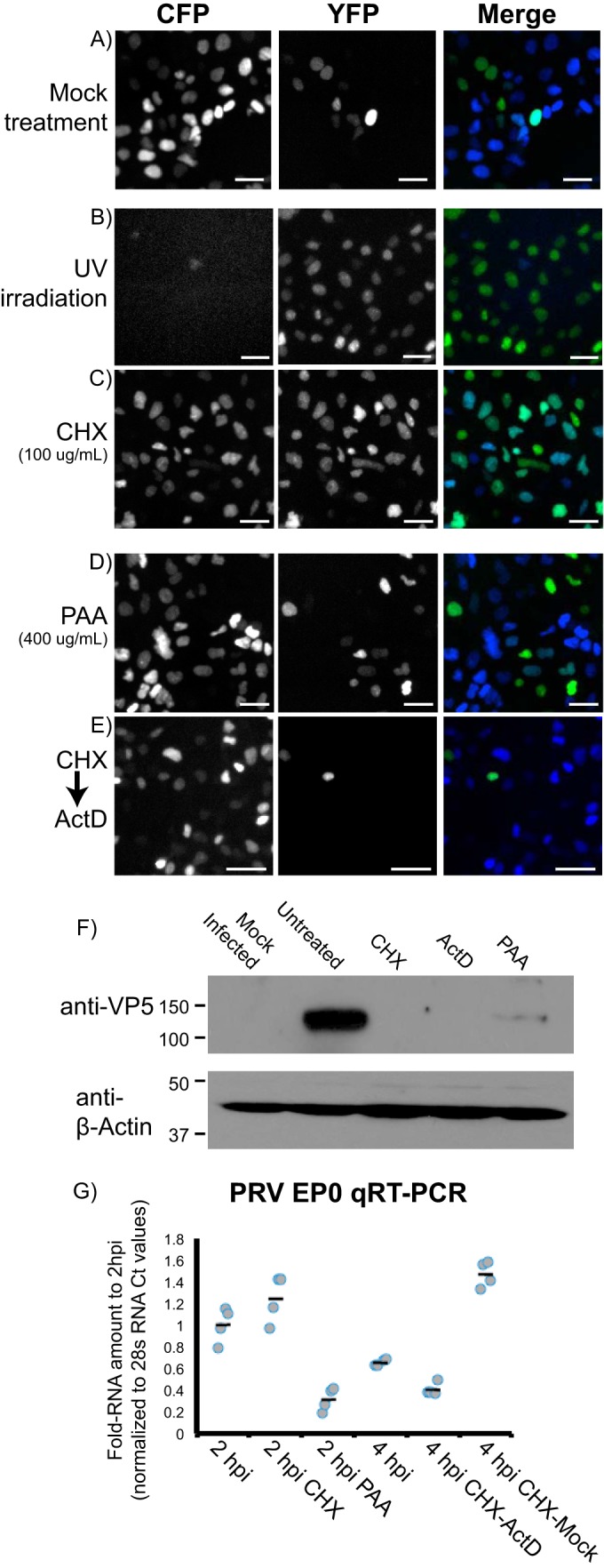
Chemical inhibition of PRV coinfection. Microscopy of *T*2 coinfections using PRV 289 (primary inoculum; MOI of 10) and PRV 287 (delayed inoculum; MOI of 10). Cells were imaged for CFP (monochrome channel, left) and YFP (monochrome channel, middle) at 6 to 8 hpi. Also depicted is a merged image of the two channels (CFP is blue, YFP is green). (A) Mock-treated cells. (B) Primary inoculum subjected to UV irradiation. (C and D) Cells treated with CHX at 100 μg/ml (C) or PAA at 400 μg/ml (D) for 2 h between the primary and secondary infections. (E) Following primary infection, cells were treated sequentially with CHX and ActD at 100 μg/ml for 2 h each before application of the PRV 287 at T4. (F) Western blot analysis of VP5 expression from PRV-infected cells during chemical treatment. Cells were either mock infected or infected with PRV 289 at an MOI of 10. Following infection, cells were mock treated or treated with CHX, ActD, or PAA as previously described. Cells were harvested at 6 hpi, and proteins were subjected to Western blot analysis with anti-VP5 or anti-beta-actin antibodies. (G) Quantitative real-time PCR analysis of viral gene transcription. Cells were infected with PRV 289 and subjected to CHX, PAA, and ActD treatment as previously described. Cells were harvested at 2 or 4 hpi, and RNA was differentially extracted and analyzed by qRT-PCR with primers to detect PRV EP0 transcripts. Relative threshold cycle (*C_T_*) values for each sample were calculated and then normalized to 28S rRNA controls. Fold change values are relative to values for 2-hpi samples (relative quantitation [Rq] values) for replicate samples (gray dots) along with average relative Rq values (black bars).

To understand the role of viral replication in establishing superinfection exclusion, we altered infection through four mechanisms: light-induced virion inactivation or cycloheximide (CHX), phosphonoacetic acid (PAA), or actinomycin D (ActD) treatment. To identify the treatment effects on superinfection exclusion, we used PRV recombinants under staggered infection conditions (*T*2). All infections were analyzed by fluorescence microscopy to visualize infection-associated FP expression between 6 and 8 h postinfection (Fig. 3).

Virion inactivation was performed on the primary viral inoculum, PRV 289, using UV irradiation or neutral red treatment, followed by white light inactivation. UV irradiation produces extensive nucleic acid cross-linking, preventing viral transcript production following virion entry (36). Similarly, neutral red intercalates into viral DNA, generating chemical adducts upon stimulation with broad-spectrum light (37). Cells first infected with inactivated PRV 289 and then infected with PRV 287 demonstrated no CFP but strong YFP expression in all cells (Fig. 3B). While neutral red is more effective at inactivating virions, it does limit the damage to nucleic acids, whereas UV irradiation can result in protein cross-linking that reduces the ability of virions to mediate entry. In conclusion, inactivation by both processes eliminates the capacity for primary viral infections to exclude the expression of FPs associated with subsequent inoculations, indicating viral entry and fusion are not sufficient to establish superinfection exclusion.

To distinguish between the need for viral protein synthesis and viral genome duplication, we utilized CHX and PAA. CHX, a ribosome translocation inhibitor, blocks new protein synthesis following viral infection. PAA, an inhibitor of the herpesvirus polymerase, inhibits viral genome duplication, delaying the expression of viral late proteins but not immediate-early or early proteins (39, 42). Following primary inoculation with PRV 289, cells were treated with either CHX or PAA for 2 h. At the 2-h time point, drug treatment was washed out and cells were subjected to secondary infection with YFP-expressing recombinants. Following CHX treatment, we observed CFP and YFP expression in the majority of cells under both conditions of infection (Fig. 3C). Following PAA treatment, we observed robust CFP expression and the exclusion of YFP expression in most cells (Fig. 3D). These treatments suggest viral genome duplication and late viral proteins are not necessary, but the synthesis of viral and cellular proteins is required to establish superinfection exclusion.

To determine if initial RNA transcripts produced during the first 2 h of infection were sufficient to establish exclusion, we used a combination of initial CHX treatment followed by ActD treatment. ActD binds DNA, inhibiting transcription, but does not prevent already-synthesized transcripts from being translated. Using the same 2-h CHX treatment as that described above, cells were washed and treated for another 2 h with ActD, followed by infection PRV 287. When ActD is applied for 2 h following CHX treatment, we observe a loss of YFP expression in infected cells.

Identical chemical treatments also were performed with HSV-1 fluorescent protein expressing recombinants. In all cases, similar results were observed, although the enhanced superinfection exclusion of HSV-1 at 2 h postinfection blunted some effects. In particular, CHX treatment increased the number of YFP-positive HSV-infected cells but did not fully restore YFP expression to the same extent as CHX treatment during PRV infections (data not shown).

To ensure all treatments affected viral replication, control experimentation for viral titer, viral protein synthesis, and viral gene transcription was performed. For virion inactivation, the titers of irradiated viral stocks were determined for PFU content and found to be reduced by >10^5^ (approximately 1 × 10^3^ PFU/ml remaining). Neutral red inactivation was much stronger, with a total loss of infectivity following 15 min of white light exposure. CHX, PAA, and ActD were tested for the capacity to prevent or reduce VP5 (late protein) expression (Fig. 3F). CHX and ActD treatments were sufficient to prevent any detectable VP5 expression by 6 h postinfection. PAA treatment reduced but did not eliminate VP5 expression. This correlates with the strong reduction in, but not the complete blocking of, infectious titers seen with PAA treatment and is concordant with previously published descriptions of PAA's activity (39, 42). Finally, the level of PRV EP0 gene transcription was measured by qRT-PCR during the drug treatments (Fig. 3G). Surprisingly, CHX alone led to a 3-fold increase in ICP0 transcript levels compared to that of untreated controls. When actinomycin D treatment followed CHX treatment, both EP0 and ICP0 transcript levels were reduced compared to those of CHX treatment followed by mock treatment.

These data indicate the drug treatments did inhibit viral replication, protein synthesis, and gene transcription. We can conclude that virion inactivation and CHX treatment inhibited the process of superinfection exclusion for both PRV and HSV. In contrast, PAA treatment and sequential CHX/ActD did not interfere with superinfection exclusion. The increase in YFP expression during CHX treatment and loss of YFP expression after subsequent ActD treatment suggests protein synthesis of transcripts produced during the first 2 h of infection is required to establish superinfection exclusion. Whether these transcripts are only the viral immediate-early genes or if some cellular proteins are needed to implement superinfection exclusion remains to be determined.

**FIG 4:**
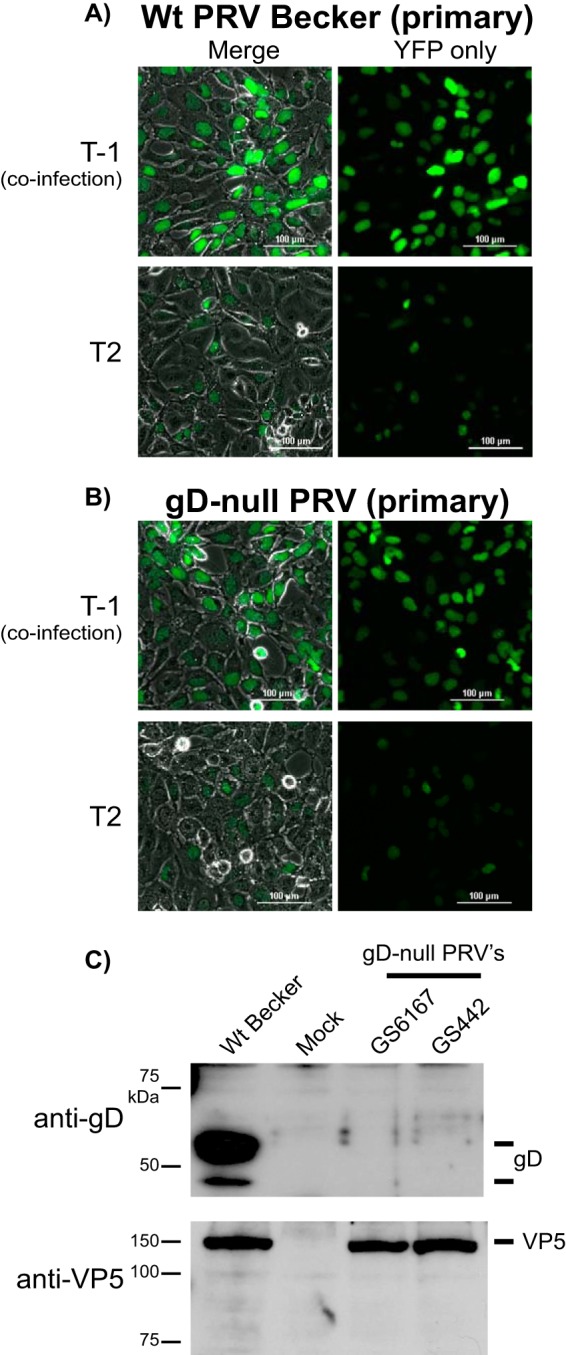
Dependence of superinfection exclusion on gD expression. PK15 cells were infected with wild-type (Wt) PRV Becker (A) or PRV GS6127, a gD-null virus, and coinfected with YFP-expressing PRV 287 (B). Viruses were applied simultaneously under *T*−1 conditions, or PRV 287 was applied at 2 hpi of the initial inoculum under *T*2 conditions. At 6 hpi, cells were imaged by phase contrast and YFP fluorescence illumination. (Left) Phase and YFP merged images. (Right) YFP alone. (B) PK15 cells were either mock infected or were infected with PRV Becker, PRV GS6127, or PRV GS442. The expression of gD or the major capsid protein VP5 was detected from infected cell extracts by Western blotting.

Previously published observations correlate superinfection exclusion to surface expression of gD, mediating a receptor interference exclusion of secondary inoculum. To determine if new gD protein expression is required, we tested the superinfection exclusion capacity of two PRV gD-null recombinants. Both PRV GS442 and GS6162 recombinant viruses contain genetic lesions in the viral genome and fail to express gD upon infection. GS442 contains a GFP insertion in the gD locus (33), whereas GS6162 has a full deletion of the gD open reading frame. Western blot detection of gD expression confirmed both viruses produced no detectable amounts of gD upon the infection of noncomplementing cells (Fig. 4B). To test superinfection exclusion, GS6162 or PRV Becker (wild-type) was used to infect PK15 cells at an MOI of 10. A second YFP-expressing virus was coinfected into cells 2 h after primary inoculation. Six hours later, infected cells were imaged for YFP expression. We observed that both PRV Becker and GS6162 prevent YFP expression from a secondary virus delayed 2 h postinfection. Similar results were obtained for GS442 and PRV 151, both expressing diffusible GFP, using an RFP-NLS FP-expressing PRV for secondary inoculation (data not shown). From these results, we conclude the exclusion of FP markers of secondary infection is independent of FP expression from the primary inoculum. More importantly, we conclude that new gD expression is not required to establish the superinfection exclusion of secondary viral inoculum.

**FIG 5:**
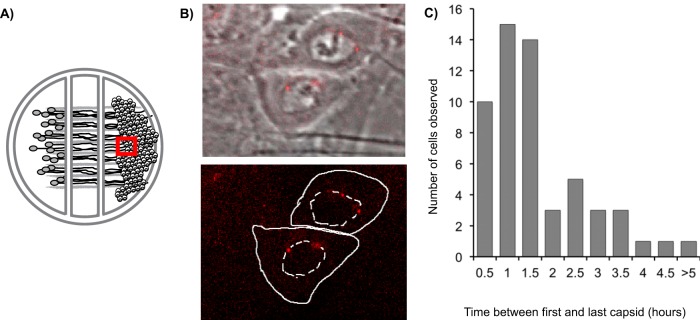
Coinfection during axon-to-cell spread. (A) Diagram of the compartmentalized neuronal cultures. The red box indicates the relative area where images were acquired. (B) Two micrographs of PK15 cells with mRFP-labeled capsids accumulating on the nuclear envelope. The top image is a phase-contrast and RFP merge. The bottom image is the RFP channel alone, with the outline of the cell border and nucleus (dashed) outlined in white. (C) Out of 153 infection events imaged, 57 cells were infected by more than 1 virion. The times between detection of the first and last capsid were calculated and cells were grouped in 30-min intervals.

We next wanted to determine if the 2-h window of superinfection correlated with the timing of coinfection during axon-to-cell spread of PRV in compartmentalized neuronal cultures. The compartmentalized neuronal cultures utilize embryonic rat SCG neurons grown under a Teflon ring that separates neuronal cell bodies from axon termini (Fig. 5A) (19, 43). Following the infection of the neuronal cell bodies, progeny virions transport within and egress from distal axons to infect susceptible cells cultured in the far compartment. The timing of virion delivery in this system is asynchronous, with the axonal spread of infection occurring between 12 and 24 h (unpublished observation). We assessed the timing of virion accumulation in susceptible cells following axonal transmission by utilizing live-cell, time-lapse imaging of compartmentalized neuronal cultures (Fig. 5A). For these experiments, neuronal cell bodies were infected with PRV 427, a recombinant virus that expresses an mRFP-VP26 fusion resulting in fluorescently labeled capsids that traffic in axons and accumulate in newly infected cells (19, 44). Utilizing time-lapse microscopy, we visualized the accumulation of capsids in susceptible cells and could determine the timing of capsid acquisition (Fig. 5B; also see Movie S1 in the supplemental material). From a cohort of 153 infection events observed, 56 events involved multiple capsids accumulating in infected cells. From the time-lapse data, we determined the time at which each capsid was detected within the infected cell and calculated the time between capsid detections. The majority of coinfection events (greater than 70%) happen within the first 2 h following the detection of the first capsid. Limited numbers of coinfection events happened at later times, up to 5 h after initial capsid detection (Fig. 5C). The distribution of coinfection times suggests cellular superinfection exclusion is part of the restriction of axon-to-cell spread of neuroinvasive alphaherpesvirus infections.

## DISCUSSION

We have utilized fluorescent protein-expressing recombinants of HSV and PRV to establish that both viruses exclude a secondary viral infection at 2 h after initial infection. This exclusion limits the expression of FPs associated with a second virus, along with associated viral progeny, from the population of infected cells. The exclusion we have characterized requires protein expression from viral and cellular transcripts produced early in infection and is independent of previously characterized gD-mediated mechanisms of receptor interference. We also have evidence, from an *in vitro* compartmentalized neuronal culture system, that superinfection exclusion may impact axon-to-cell spread of infection.

Previous work has identified the presence of a superinfection exclusion mechanism for autologous (same virus) and heterologous (different but related) alphaherpesvirus infection (29). Most of these studies have attributed the mechanism to a gD-mediated process occurring between 4 and 8 h postinfection (22, 27). We are the first to demonstrate exclusion is dependent on immediate-early protein synthesis and independent of gD-mediated receptor interference. Our results also demonstrate superinfection exclusion is present with similar kinetics between two divergent alphaherpesviruses and is cell type independent. Our own observations are supported by previous evidence finding the exclusion of a secondary viral inoculation at 2 h postinfection (26, 30). Banfield et al. characterized FP expression exclusion with similar kinetics in cultured DRG neurons during coinfections with recombinant PRVs derived from the vaccine strain Bartha, whereas Meurens et al. saw a reduction in recombinant production and coinfection of equine herpesvirus beginning at 2 h postinfection that took longer to fully exclude secondary viral infections.

The mechanism of 2-h superinfection exclusion for alphaherpesvirus infection currently is unknown. The chemical inhibitor studies reduce the potential list of viral proteins to the immediate-early class of viral genes. Thus, a first analysis suggests viral genes that mediate viral superinfection exclusion are ICP0, ICP4, ICP22, and ICP27. Only ICP4 and ICP27 share significant homology between HSV and PRV. The loss of PRV IE180 (ICP4) expression ablates viral immediate-early and late gene expression and allows a second virus to superinfect cells and neurons (45). In contrast, HSV-1 mutants lacking ICP4 (the IE180 homolog) found some immediate-early gene transcription (46), although superinfection capacity was untested. It is possible that superinfection exclusion evolved in parallel between HSV and PRV, such that nonhomologous protein sequences mediate similar effects. Additionally, there is the potential that the limited expression of the early class of viral genes and cellular proteins also are involved in establishing superinfection exclusion. Interestingly, experiments using 4-thiouridine labeling and next-generation sequencing have found evidence for the transcription of a wide range of viral genes at early times postinfection (47). Future experiments utilizing differential viral gene knockouts will identify the viral proteins and cellular processes needed to implement superinfection exclusion.

We do not know which step of viral infection is impeded during superinfection exclusion, be it virion attachment, entry, genome delivery, or another later step. Our results demonstrating 2-h exclusion occurs independently of gD expression suggest secondary virions still engage cellular receptors. Our analysis of axon-to-cell spread visualizes labeled capsids accumulating on the nuclear envelope of infected cells. Virions blocked and accumulating postentry, but before nuclear pore engagement, would not be reliably detected. Unique mechanisms of superinfection exclusion have been observed with diverse viruses. Poxviruses express proteins to repel incoming virions as well as prevent membrane fusion required for entry (3, 4). Alternatively, RNA viruses, including West Nile and Sindbis viruses, allow secondary virion entry but suppress the formation of replication complexes (5, 7). Further experimentation using targeted viral gene deletions and fluorescently labeled virions may elucidate the stage during HSV-1 and PRV infection that establishes superinfection exclusion.

Understanding the timing of superinfection exclusion is important to understanding how populations of viruses interact during infection within a host. Defining host interactions during the infection of alphaherpesviruses is complicated by the role of latency, wherein viral genomes are maintained in a quiescent, episomal state. Previous studies have indicated that latency transcripts (LAT) play a role in exclusionary mechanisms of infection (48). While it is possible an early burst of LAT transcript is sufficient to prevent a second virus from participating in infection, our findings regarding the necessity of protein synthesis suggest an RNA transcript is insufficient to establish exclusion in our experiments. A second aspect to *in vivo* infections is the route of neuroinvasion. Infections of peripheral ganglia require retrograde spread of virus from the axon terminus to the neuronal cell body. A recent report utilizing retrograde coinfections of isolated axons demonstrate that virions activate axonal injury pathways to facilitate long-distance transport (49). In contrast to our own data, Koyuncu et al. found UV-inactivated virions are capable of excluding infectious virus, suggesting a competitive interference of defective particles for retrograde transport machinery in axons. Combined with our data that superinfection exclusion impacts the spread of virions out of axons, it appears that alphaherpesvirus infections are impacted by multiple restrictions to coinfectivity during neuronal spread.

The overall influence of superinfection exclusion on pathogenic neuroinvasive viral infections has yet to be determined. Future studies will determine if these restrictions have a detrimental role in viral fitness and viability or the ability of the virus to avoid immune control. Further work will identify the viral proteins that activate exclusion, which then can determine the importance of superinfection exclusion on viral replication and disease.

## Supplementary Material

Supplemental material
