# Supplementary material for: gD-Independent Superinfection Exclusion of Alphaherpesviruses
Source: J Virol. 2016 Mar 28;90(8):4049–58. doi: 10.1128/JVI.00089-16 (PMC4810564; doi:10.1128/JVI.00089-16)
Supplement: Supplemental material [file supp_90_8_4049__index.html]

gD-Independent Superinfection Exclusion of Alphaherpesviruses — Supplemental material 

# gD-Independent Superinfection Exclusion of Alphaherpesviruses

## Supplemental material

- Supplemental file 1 -

  Movie S1 (Live-cell imaging of mRFP-VP26-labeled capsids during anterograde spread of infection.)

  AVI, 1.8M
- Supplemental file 2 -

  Supplemental Movie legend.

  PDF, 47K
