# Supplementary material for: gD-Independent Superinfection Exclusion of Alphaherpesviruses
Source: J Virol. 2016 Mar 28;90(8):4049–58. doi: 10.1128/JVI.00089-16 (PMC4810564; doi:10.1128/JVI.00089-16)
Supplement: Supplemental material [file JVI.00089-16_zjv999091542so2.pdf]

Supplementary Movie 1: Live cell imaging of mRFP-VP26 labeled capsids during anterograde spread of infection.

Compartmentalized SCG neuronal cultures were infected with PRV 427. Beginning 5 hours post infection, PK15 cells seeded into the axon compartment were imaged every five minutes with phase contrast and for RFP and YFP fluorescence. The displayed movie is a cropped section of a larger image tile acquired during sequential imaging. The center of the image has two cells that acquire multiple mRFP puncta, indicative of labeled capsids, prior to the onset of YFP expression. The movie has been compressed to display 5 frames per second.
